# Application of Topical Hyperbaric Oxygen Therapy and Medical Active Dressings in the Treatment of Arterial Leg Ulcers—A Pilot Study

**DOI:** 10.3390/s23125582

**Published:** 2023-06-14

**Authors:** Jarosław Pasek, Sebastian Szajkowski, Grzegorz Cieślar

**Affiliations:** 1 Collegium Medicum im. dr Władysława Biegańskiego, Jan Długosz University in Częstochowa, 13/15 Armii Krajowej St., 42-200 Częstochowa, Poland; 2 Faculty of Medical and Social Sciences, Warsaw Medical Academy of Applied Sciences, 8 Rydygiera St., 01-793 Warszawa, Poland; sebastianszajkowski@wp.pl; 3 Department of Internal Medicine, Angiology and Physical Medicine, Faculty of Medical Sciences in Zabrze, Medical University of Silesia in Katowice, 15 Stefana Batorego St., 41-902 Bytom, Poland; cieslar1@o2.pl

**Keywords:** arterial leg ulcers, treatment, specialized medical dressings, local hyperbaric oxygen therapy, chronic wounds

## Abstract

Leg ulcers are a very serious worldwide medical problem. When the ulcer is extensive and deep the prognosis is usually unfavorable. The treatment requires comprehensive solutions that take into account modern specialized medical dressings, and more and more often, selected methods in the field of physical medicine. The study included 30 patients (13 women—43.4% and 17 men—56.6%) with chronic arterial ulcers of the lower limbs. The mean age of the treated patients was 65.63 ± 8.77 years. Patients were randomly assigned to two study groups. In group 1 (16 patients), specialist ATRAUMAN Ag medical dressings and local hyperbaric oxygen therapy treatments were used. In group 2 (14 patients), only specialized ATRAUMAN Ag dressings were used. The treatment was carried out for 4 weeks. The progress of healing ulcers was assessed by using the planimetric method, while the intensity of pain ailments was assessed by the visual analog VAS scale. In both study groups, a statistically significant reduction in the mean surface area of the treated ulcers was obtained, respectively, from 8.53 ± 1.71 cm^2^ to 5.55 ± 1.11 cm^2^ in group 1 (*p* < 0.001) and 8.43 ± 1.51 cm^2^ to 6.28 ± 1.13 cm^2^ in group 2 (*p* < 0.001). There was also a statistically significant reduction in the intensity of pain ailments, respectively: 7.93 ± 0.68 points to 5.00 ± 0.63 points in group 1 (*p* < 0.001) and 8.00 ± 0.67 points to 5.64 ± 0.49 points in group 2 (*p* < 0.001). The percentage change in ulcer area from baseline in group 1 was 34.6 ± 8.47% and was statistically significantly greater than in group 2 (25.23 ± 6.01%) (*p* = 0.003). In turn, the percentage assessment of the pain intensity assessed in the VAS scale in group 1 was 36.97 ± 6.36% and was statistically significantly higher compared to group 2 (29.34 ± 4.77%) (*p* = 0.002). The addition of local hyperbaric oxygen therapy treatments as a supplement to the therapy with the use of specialized medical dressings improves the effectiveness the arterial ulcers treatment of the lower limbs in terms of reducing the ulceration area and reducing pain ailments.

## 1. Introduction and Background

Leg ulcers, regardless of their etiology, require urgent and multidirectional therapeutic treatment in each case. Patients with lower limb ulcers are at a very high risk of complications from the vascular system and the consequent need to amputate the limbs [1,2].

The most common type of chronic leg ulcers is venous ulcers caused by excessive pressure in the venous system and an insufficiency of the venous valves. Arterial ulcers are next in frequency, with atherosclerosis being the most common cause. The disease is diagnosed annually in about 40.000 patients. The incidence of atherosclerosis is four times higher in men than in women and the clinical symptoms of atherosclerotic changes are most often visible between 50 and 60 years of age. Despite the intensive development of medicine, the etiopathogenesis of this disease is still not fully understood [3,4].

The Incidence of arterial ulcers of the lower limbs is estimated in Europe and the United States at 500–1000 new cases per million inhabitants per year, and their incidence is 1.3–2.3% of the population [5]. Due to the aging of societies, more frequent lower limb ischemia, and the application of increasingly accurate diagnostic methods, the frequency of ulceration detection has increased [5,6,7]. The burden on the health service for the treatment of lower limb ulcers, including patients with peripheral arterial disease, is significant. Reducing these burdens through the use of alternative forms of treatment can bring measurable benefits and financial savings [5].

Atherosclerotic changes cause difficult blood flow in blood vessels and hemodynamic disorders. One of the three main clinical manifestations is atherosclerotic ischemia of the lower limbs, which, in the advanced stage of the disease, may take the form of critical ischemia of the lower limbs. The basic cause is usually the occlusion of large arteries, which gradually reduces the blood supply to the peripheral structures of the limbs at rest, as a result of which, their supply of nutrients is significantly reduced, causing very severe pain, a numbness of the limbs, and difficulty in moving. A reduced blood perfusion leads to changes in the microcirculation and is manifested by foot pain, trophic changes in the skin, non-healing ulcers, or tissue gangrene [1,8].

The basic methods of the atherosclerosis treatment of the lower limbs are in fact vascular revascularization and endovascular procedures [9,10,11].

In the last twenty years, there have been reports in the literature on the use of innovative non-pharmacological methods on the treatment chronic wounds, also used in the treatment of leg ulcers. In addition to the outer cover of wounds, these methods stimulate the natural processes of tissue regeneration, provide the right environment for the healing process, and protect the inside of the wound against the development of infection. Other (alternative) methods of therapy include negative pressure wound therapy (NPWT)—which is also increasingly used—platelet-rich plasma and cellular growth factors, selected methods of physical medicine (low-energy lasers, high-voltage electrostimulation, magnetotherapy, magnetostimulation, ultrasound, deep oscillation treatments, vibroacoustic therapy, ozone therapy), and dressings containing silver with antibacterial and antifungal properties. As a result of the content of active ingredients, such as absorption gels or sodium hyaluronate, modern dressings also create optimal conditions around wounds, favoring the intensification of the healing process. They also play an important role in wound cleaning. Wound cleaning is a very important element of the therapy that affects the course of the healing process. This requires carrying out specific procedures or treatments related to their cleaning, exudating removal. Modern dressings used in the treatment of ulcers are classified as so-called active dressings, i.e., those that support the healing process of ulcers. Firstly, modern dressings maintain a moist wound environment, optimize the level of exudate typical of chronic wounds (balance the enzyme/inhibitor ratio, reduce the concentration of pro-inflammatory cytokines, increase the availability of growth factors and nutrients in the wound), protect the wound from bacterial access, and ensure proper exchange of gaseous and optimal constant temperature without increasing the risk of infection. Such conditions are conducive to biological processes and facilitate cell division, migration, and differentiation, regulate enzymatic activity, and accelerate the reconstruction of protective layers of the epithelium, stimulating granulation. It is also important to isolate and protect the wound against the harmful effects of external factors. The dressing may adhere to the wound surface, but it should be easy to remove from the wound (without damaging the newly formed granulation tissue). It should also have sorption properties and prevent secondary wound infections. Currently, the raw material used in specialist dressings is very often ionic silver with a documented strong bactericidal effect [1,2,12,13,14,15,16,17].

Among the physical methods used in the treatment of wounds of various etiologies there are also hyperbaric oxygen therapy procedures (in addition to those mentioned above) [12,18,19]. Hyperbaric oxygen therapy (HBOT) is a therapeutic method that uses the therapeutic effect of 100% oxygen or a mixture of gases with a high oxygen content close to 100% at a pressure above 1 atmosphere. HBOT treatments are performed in single or multi-seat chambers and consist of the administration of pure oxygen at an increased pressure for breathing or the direct impact of hyperbaric oxygen on the surface of the patient’s body. In the conditions of systemic hyperbaric oxygen therapy conducted in hyperbaric chambers, the oxygen pressure in the alveolar air increases in accordance with Henry’s gas law, as a result of which, after crossing the alveolar–capillary barrier, it enters the pulmonary bloodstream in an increased amount. Hyperbaric oxygen therapy improves metabolism in the wound by stimulating the proliferation of fibroblasts and the formation of a collagen framework needed for proper angiogenesis processes. It also affects the stimulation of growth factors involved in the wound healing process. Oxygen also has an antibacterial and antifungal effect by activating leukocytes, inhibiting the proliferation of anaerobic bacteria and the production of bacterial toxins, and supporting the penetration of antibiotics [20,21].

A few years ago, hyperbaric chambers for the local application of oxygen therapy appeared on the medical market, which, while retaining all the advantages of systemic hyperbaric oxygen therapy, ensure comparable therapeutic effectiveness, and at the same time, guarantee a greater safety of therapy as they are devoid of typical side effects occurring in the case of systemic hyperbaric oxygen application. Local hyperbaric oxygen therapy also allows the treatment of those patients in whom systemic hyperbaric oxygen therapy is contraindicated for many reasons [22].

The aim of the study was to compare the therapeutic efficacy of combined treatment, including medical active dressings and topical hyperbaric oxygen therapy versus medical active dressings applied alone in the treatment of arterial leg ulcers.

## 2. Material and Methods

The study included 30 patients (13 women and 17 men) aged 47–80, hospitalized in the Department of Internal Medicine, Angiology and Physical Medicine in Bytom with a diagnosis of arterial ulceration of the lower limbs, who had no indications for vascular revascularization.

After collecting their medical history and performing a physical examination, the patients were randomly (one-to-one) assigned to two study groups. Group 1 (16 patients) was treated with specialist ATRAUMAN Ag dressings and local hyperbaric oxygen therapy procedures. In group 2 (14 patients), only specialized ATRAUMAN Ag medical dressings were used. The treatment was carried out for a total of 4 weeks.

The use of ATRAUMAN Ag dressings was dictated by the current recommendations for their use. It is a specialist dressing that does not stick to the wound, and thus allows for its painless removal, which is not without significance in the case of coexisting pain. In addition, these dressings provide good ventilation and oxygenation of the wound, which accelerate healing and minimizes the risk of complications. In addition, the constant release of silver ions into the wound bed stabilizes bacterial activity at a low level, and the creation and maintenance of an optimal moist wound environment stimulates the autolysis of necrosis and the formation of new granulation tissue.

The mean age of patients in group 1 (66.31 ± 7.7 years) did not differ statistically significantly as compared to group 2 (64.85 ± 10.09 years) (*p* = 0.867). Additionally, the mean value of the ABI index in group 1 (24.07 ± 1.41 kg/m^2^) did not differ statistically significantly as compared to group 2 (24.91 ± 2.60 kg/m^2^) (*p* = 0.924). The mean time of ulceration occurrence in group 1 was 3.53 ± 0.42 years and in group 2, 3.31 ± 0.86 years, respectively. The difference was also not statistically significant (*p* = 0.727).

The ulceration conditions in both groups were comparable. The ulcerations were located above the lateral or medial malleolus. The surface of the wounds was usually covered with pale yellow fibrin with single necrotic hyphae. The wounds were characterized by atrophic changes. The skin around the ulcerations was parchment-like, shiny, with an unpleasant odor, a slight exudation, and an admixture of purulent secretion. In both groups, the ulceration area and depth (small shallow ulcer on distal leg, no exposed bone, no gangrene), incidence of infection (mild clinical manifestation of infection; local swelling and opaque to white secretion) and ABI index values (0.6–0.79) were all, in both groups, in grade 1, according to the WIfl Classification System [23]. All qualified patients have ulcerations that qualified for the applied dressing material.

The inclusion criteria for the study were as follows: arterial leg ulcers of the lower limbs, age ≥47 and ≤80 years, lack of qualification for vascular revascularization, and voluntary patient consent to participate in the study. Exclusion criteria from the study were as follows: etiology of ulcers other than arterial, deep vein thrombosis, acute ischemia of the lower limbs, qualification for vascular vascularization, age <47 and >80 years, generalized infection, contraindications to hyperbaric oxygen therapy treatments, allergy to silver, and a lack of patient consent to participate in the study.

Prior to the treatment implementation, each patient from both study groups underwent a Doppler ultrasound examination (in order to determine the etiology of the ulceration). A surgical consultation was also performed in each patient. In the case of necrotic tissue or purulent infiltration within the ulceration, surgical wound debridement was performed before inclusion to the study.

Specialized dressings were changed every three days in both study groups. The dressings used in the study had the properties to ensure a moist environment conducive to accelerating the healing of ulcers. They did not require daily replacement and at the time of their change, they did not damage the newly formed granulation tissue. The only contraindication to their use during the studies was the previously diagnosed allergy of patients to the active substance contained in them (silver ions).

**Figure 1:**
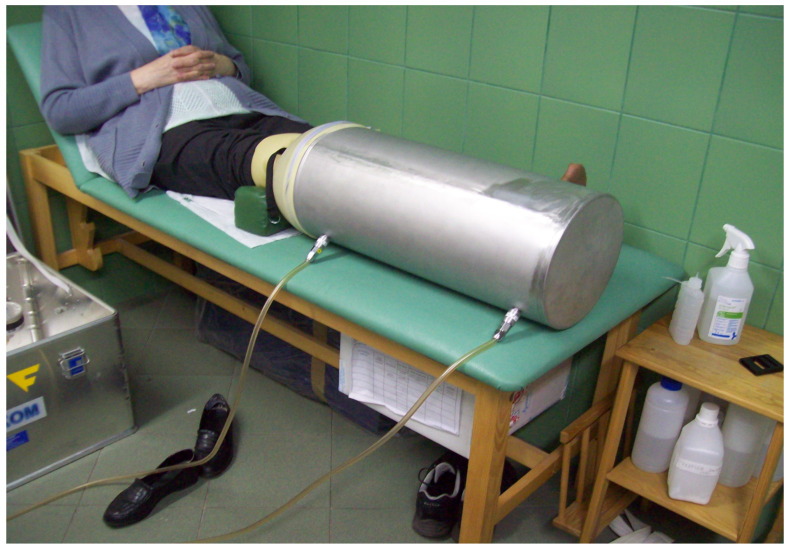
The procedure of local hyperbaric oxygen therapy with the use of OXYBARIA-S device (manufacturer Firma FASER S.A., Świętochłowice, Poland).

The OXYBARIA-S device (manufacturer Firma FASER S.A., Świętochłowice, Poland) was used for local hyperbaric oxygen therapy [24]. Treatments were performed for four weeks, every day from Monday to Friday, with a Saturday–Sunday break. Each procedure lasted 30 min. During HBOT treatments, the treated limb of the patient was placed in an aluminum cylindrical applicator into which after sealing at the thigh level with a silicone collar, oxygen was introduced from an external cylinder with a concentration of approx. 95%, pressure 1.5 mBa, and flow about 5 L/min (Figure 1).

During the treatment, various forms of standard pharmacotherapy were used (pentoxifylline, sulodexide, and local antiseptic therapy). No antibiotics were administered before or during the evaluation period.

The study protocol was approved by the local bioethics committee of the Medical University of Silesia in Katowice (approval number: KNW/0022/KB1/102/II/16/19). Each patient signed a written informed consent to participate in the study.

### 2.1. Planimetric Assessment of Ulcer Surface Area

In clinical practice, modern measurement methods are used to assess the effects of wound treatment using computer programs for the digital processing of images used for image processing. They are advanced techniques of non-contact planimetry characterized by high reliability and an accuracy of measurements. They do not require direct contact with the wound, which reduces the risk of its contamination and damage to the treated tissues.

Author’s computer software was used to assess the surface of the treated ulcers by a planimetric measurement of the surface area in manual mode [25]. In the first stage, a doctor loaded the analyzed picture from a digital photo of a leg ulcer, with a subsequent selection of ulcer contours, by moving the mouse cursor continuously along the contour of the target area. Next, double-clicking the mouse cursor resulted in automatically closing the drawn contour and creating a closed curve. When the selection stage was completed, the software automatically calculated the surface area within the previously defined contour. The surface area was represented in pixels and after adequate calculation in square centimeters. In order to maintain high accuracy, all planimetric measurements were performed by only one specialist experienced in this method. The method used allows for calculating the absolute value of the wound surface area and enables systematic monitoring and verification of the healing progress by comparing the results of wound surface area measurements at specific time intervals [25].

### 2.2. Measurement of Pain Intensity

Before and after the end of the treatment, the intensity of pain ailments was assessed with the use of the ten-point visual analog scale (VAS) for a subjective assessment of pain intensity. In this method, the patient marks a section of a straight line, usually 10 cm long, with opposite ends marked, and a 1 cm scale, a point that corresponds to the intensity of pain experienced by him—from 0, which means no pain at all, to 10, which, in simple terms, indicates unbearable pain (the strongest pain the patient has ever felt in their life) [26].

### 2.3. Statistical Analysis

Statistica 13 software (Statsoft, Poland) was used for statistical analysis. The Shapiro–Wilk test was used to test the normality of data. The statistics is presented as mean, standard deviation (SD), and 95% confidence intervals (CI). The Mann–Whitney U test was used to compare two unmatched groups. The Wilcoxon test was used to compare two matched groups of data. Statistical significance level was *p* < 0.05.

## 3. Results

Before treatment, the mean ulceration area in group 1 (8.53 ± 1.71 cm^2^) was not statistically significantly different from the mean ulceration area in group 2 (8.43 ± 1.51 cm^2^) (*p* = 0.851). As a result of the applied treatment, the mean value of the ulceration area in both groups was statistically significantly reduced: from 8.53 ± 1.71 cm^2^ to 5.55 ± 1.11 cm^2^ in group 1 (*p* < 0.001) and from 8.43 ± 1.51 cm^2^ to 6.28 ± 1.13 cm^2^ in group 2 (*p* < 0.001), respectively. After the treatment, the mean values of ulceration area in both groups did not differ statistically significantly (*p* = 0.124) (Table 1).

Before the treatment, the average value of the pain intensity in group 1 (7.93 ± 0.68 points) did not differ statistically significantly from the mean value of the ulcer surface area in group 2 (8.00 ± 0.67 points) (*p* = 0.819). As a result of the treatment, the average value of pain intensity in both groups was statistically significantly reduced: from 7.93 ± 0.68 points to 5.00 ± 0.63 points in group 1 (*p* < 0.001) and from 8.00 ± 0.67 points to 5.64 ± 0.49 points in group 2 (*p* < 0.001), respectively. After the treatment, the mean VAS pain intensity values in both groups did not differ statistically significantly (*p* = 0.015) (Table 2).

The percentage improvement after the applied treatment was also analyzed in terms of reducing the surface area of ulcers and pain. In group 1, the area of treated ulcers decreased on average by 34.6 ± 8.47%, and in group 2 by 25.23 ± 6.01%. The observed difference was statistically significant (*p* = 0.003). In group 1, the intensity of pain assessed using the VAS scale decreased on average by 36.97 ± 6.36%, and in group 2 by 29.34 ± 4.77%. The observed difference was also statistically significant (*p* = 0.002) (Table 3).

All examined patients completed a full therapeutic cycle and did not report any side effects related to the treatment during and after the therapeutic cycle.

## 4. Discussion

The knowledge of the use of various treatment methods in the case of hard-to-heal wounds, including leg ulcers, is systematically and continuously expanded and updated. The modern concept of wound treatment involves a comprehension of the underlying disease and is aimed at creating conditions conducive to healing, e.g., by eliminating risk factors responsible for the abnormal course of healing such as obesity, diabetes, malnutrition, hyperlipidemia or nicotinism [8,14,15,27]. According to the latest guidelines and the European Wound Management Association, the treatment of hard-to-heal wounds should be carried out in accordance with the TIMERS strategy (T—tissue management, I—infection and inflammation control, M—hydration balance, E—epithelial advancement, R—repair and regeneration, and S—social and social individual factors) [14,15,28]. Dissemond believes that the effective treatment of patients with chronic leg ulcers is possible, but only after a thorough examination of the patient and the appropriate diagnosis. Currently, the therapeutic possibilities are wide, including conservative and surgical treatment [29]. In turn, Hedayati et al., based on the analysis of more recent studies, indicate that there is no consensus on the optimal treatment modality, whether conservative or operative [30].

It should be emphasized that in patients with complications of peripheral arterial disease—also in the form of arterial leg ulcers—the most important treatment is vascular revascularization, which enables the restoration of a possibly physiological blood supply. In patients who are not eligible for vascular revascularization for various reasons, recommended and effective methods should be implemented, among which, dressings are considered the standard of local treatment; however, there is a rise in innovative methods in the field of physical medicine (physiotherapy), which could also be effective. This comprehensive, coordinated approach, while taking into account various methods of action, is intended to accelerate the healing process, facilitate the cleansing of necrotic tissue from ulcers, reduce exudation and swelling, and support granulation processes. However, the effects of the treatment of hard-to-heal wounds are still not fully satisfactory, as they often do not ensure complete wound healing [3,31]. Moreover, according to Broderick et al., currently, due to the low number of scientific publications, there is insufficient evidence to indicate which topical agents or appropriate dressings should be used, thus influencing the treatment of arterial leg ulcers [16].

Currently, the greatest hopes are associated with dressings that contain active substances. The developed concept of an “ideal” dressing assumes that it should not only have a defensive function and provide a sufficiently moist environment, but also directly stimulate cell regeneration [8,13,28].

Eleven specialists in the management of various types of wounds discussed best practices, recommendations, and guidelines for the management of the main types of chronic wounds. An analysis of the collected literature showed that significant progress has been made in the understanding of wound healing processes over the past decade. There have also been many publications emphasizing the importance of medical dressings and various forms of therapy. Experts also point out that more emphasis should be placed on making the right diagnosis and choosing the right dressing and appropriate therapy. Such a holistic approach to the treatment of the patient and the wound bed is aimed at achieving the best therapeutic result while preventing the recurrence of wounds [6].

Bowers and Franco, however, disagree. These authors believe that patients with arterial ulcers should be immediately referred to a vascular surgeon who should consider appropriate intervention [3]. They came to a similar conclusion to Cooke et al., who also believe that the treatment of limb-related conditions is best addressed with surgical and endovascular interventions. According to the authors, these procedures restore proper perfusion and current conservative therapies have little effect on improving blood flow in the limbs [32].

In our study, we used specialized active dressings containing silver ions, the effect of which resulted in a statistically significant reduction in the surface area of the treated ulcers, thus accelerating the healing process in both assessed groups of patients. The therapeutic effect obtained was greater in the group of patients who additionally received local HBOT treatments. It was most likely related to the synergistic therapeutic effect of simultaneously used therapies. HBOT treatments improve tissue metabolism and their increased oxygenation, which translate into higher results. In hyperbaric treatment, hemoglobin is more saturated with oxygen; the oxygen concentration dissolves physically and is not bound to hemoglobin increase in the serum, thereby resulting in significantly accelerated healing processes. The proper course of the wound healing process involving leg ulcers requires the right amount of oxygen supply to the tissues. At the stage of inflammation, oxygen affects the intensity of migration and proliferation of fibroblasts. If the supply of the appropriate amount of oxygen and nutrients involved in the tissue regeneration process is limited, the healing process is disturbed and thus significantly prolonged [18,21,33].

In the analysis conducted by Bouza et al., the authors analyzed the scientific evidence confirming the effectiveness of modern dressings used in the treatment of leg ulcers by searching the available databases. As in our case, the authors did not find any studies that focused solely on arterial ulcers. The results of the analysis showed no statistically significant differences in the percentage of healed ulcers or reduction in wound size for both modern and conventional dressings. As in the case of hyperbaric oxygen therapy treatments, it can be inferred that the current medical literature is poor in terms of research regarding this topic. There is insufficient scientific evidence to determine whether the choice of a specific type of dressing has the most beneficial effect on the course of ulcer healing [34].

In one of the older articles, the authors evaluated randomized clinical trials to determine whether applied topical agents and dressings affect the rate of healing of arterial ulcers of the lower extremities. Only one study met the inclusion criteria and was analyzed. The authors emphasize that the study included a group that was too small in number of participants to be analyzed, and the observation period was too short. On this basis, it cannot be concluded whether there was any difference in cure rates [13].

Mosti et al. compared the treatment efficacy of a microbiological binding (MB) dressing with a silver-containing hydrofiber (SCH) dressing. The study group consisted of 20 patients (15 patients with venous leg ulcers and 5 with arterial leg ulcers). An assessment was made on the bacterial load of heavily colonized or locally infected wounds. The analysis of the obtained results showed that both types of dressings are effective in reducing the bacterial load in critically colonized or locally infected chronic leg ulcers. However, MB dressings showed a higher efficiency. No adverse effects were found [35].

Unfortunately, the available literature also lacks current papers that would explicitly discuss the therapeutic benefits of HBOT in the treatment of arterial ulcers. Therefore, we could not compare our preliminary results or assess the safety of the therapy used in relation to other scientific reports.

In one of the few articles by Kranke et al. in 2015, they reviewed the available results of randomized trials, in which they tried to answer the following questions: (1) does hyperbaric oxygen therapy (HBOT) increase the percentage of patients with healed chronic wounds and (2) does the need to perform lower limb amputations subsequently decrease? Most of the studies were concerned with ulcers related to diabetes and venous diseases, which confirm the effectiveness of HBOT therapy in terms of wound healing. The authors did not find any data confirming or disproving the effect of HBOT on the healing of arterial ulcers [18].

In turn, Heyboer et al. conducted a retrospective analysis on a group of patients who received hyperbaric oxygen therapy treatments for ulcers caused by arterial insufficiency that did not heal despite standard treatment. Eighty-two patients were analyzed. The overall cure rate was 43.9% and the amputation rate was 17.1%. According to the authors, hyperbaric oxygenation may constitute a positive role in the treatment of leg ulcers of arterial etiology, which failed standard treatment [36].

Beric et al. conducted a prospective clinical trial with 80 patients. The patients were divided into two groups: (group 1) 40 patients with arterial occlusive disease and lower limb wounds, with a subgroup (n = 20) treated with HBO therapy, and (group 2) 40 patients with diabetic angiopathy and lower limb wounds, with a subgroup (n = 20) treated with HBO therapy in addition to standard therapy. In both groups, statistically significantly higher results were found in patients who underwent HBO treatments as an addition to the standard therapy [37].

The analysis of the obtained results also showed beneficial therapeutic effects related to the reduction in pain in both study groups. The mechanisms of the analgesic effect of HBOT include, among others: stimulating the secretion of endogenous endorphins and inhibiting the activity of the local inflammatory process.

John et al. summarized in their article the available scientific reports on the non-surgical treatment of arterial leg ulcers, topical therapies, pharmacological agents, apparatus and available devices. Only randomized clinical trials and meta-analyses were included. The results showed that, despite some limitations of these methods and side effects, their use should be considered in each case because these procedures are characterized as having good tolerance, accessibility, and impact on the reduction in pain ailments [38].

Taking into account the good tolerance of the treatments and the small number of contraindications to the use of local hyperbaric oxygen therapy, when used together with active specialized dressings, these treatments can be a valuable supplement to pharmacological treatment. Moreover, it should be emphasized that local HBOT can be conducted at home and is preferred for people with limited mobility who, for various reasons, cannot be treated in specialist stationary centers. Furthermore, this method reduces pain and also improves tissue oxygen perfusion.

These positive preliminary therapeutic effects should allow for the optimization of future therapeutic procedures and the refinement of the treatment of chronic arterial ulcers of the lower limbs.

### Limitations of the Study

This study had some limitations. First, this was a pilot study performed in a single institution with the risk of potential bias with respect to data collection. The study also did not include the analysis of previous standards of care used in the analyzed group of patients.

## 5. Conclusions

The use of combined therapy, including specialized dressings and local hyperbaric oxygen therapy procedures, results in a more effective reduction in the surface area of treated arterial ulcers of the lower limbs and the intensity of pain, compared to specialized dressings used as monotherapy. Taking into account the good tolerance and the small number of contraindications to the use of local hyperbaric oxygen therapy, these methods used together with active specialized dressings can be a valuable supplement to pharmacological treatment, especially for people with limited mobility and for patients who are not eligible for vascular revascularization for various reasons. It is therefore justified to conduct further multi-center studies on larger groups of patients, the results of which would enable the optimization of the analyzed therapeutic procedures.

## Figures and Tables

**Table 1:** Comparison of the average values of the surface area of treated arterial ulcers of the lower limbs between the study groups before and after treatment, together with statistical analysis.

	Ulcer Surface Area (cm^2^)				** *p* _(Before vs. After)_
	Before		After		
	Mean ± SD	(95% CI)	Mean ± SD	(95% CI)	
Group 1	8.53 ± 1.71	7.61–9.44	5.55 ± 1.11	4.95–6.14	<0.001
Group 2	8.43 ± 1.51	7.56–9.30	6.28 ± 1.13	5.63–6.93	<0.001
* *p* _(Gr.1 vs. Gr.2)_	0.851		0.124		

* Mann–Whitney U test; ** Wilcoxon test.

**Table 2:** Comparison of mean pain intensity values assessed on the VAS scale between the study groups before and after treatment with statistical analysis.

	VAS Score (Points)				** *p* _(Before vs. After)_
	Before		After		
	Mean ± SD	(95% CI)	Mean ± SD	(95% CI)	
Group 1	7.93 ± 0.68	7.57–8.29	5 ± 0.63	4.66–5.33	<0.001
Group 2	8 ± 0.67	7.60–8.39	5.64 ± 0.49	5.35–5.92	<0.001
* *p* _(Gr.1 vs. Gr.2)_	0.819		0.015		

* Mann–Whitney U test; ** Wilcoxon test.

**Table 3:** Comparison of the percentage improvement after the applied treatment between the study groups in terms of reducing the size of the ulceration area and pain with statistical analysis.

	Group 1		Group 2		* *p* _(Gr.1 vs. Gr.2)_
	Mean ± SD	(95% CI)	Mean ± SD	(95% CI)	
Percentage change of ulcer surface area after treatment	34.60 ± 8.47	30.08–39.11	25.23 ± 6.01	21.76–28.70	0.003
Percentage change of VAS score after treatment	36.97 ± 6.36	33.57–40-36	29.34 ± 4.77	26.58–32.09	0.002

* Mann–Whitney U test.

## Data Availability

The datasets used and/or analyzed during the current study are available from the corresponding author on reasonable request.
